# Comparison of Morphological and Physicochemical Properties of a Floury Rice Variety upon Pre-Harvest Sprouting

**DOI:** 10.3390/foods10040746

**Published:** 2021-04-01

**Authors:** Chae-Min Han, Jong-Hee Shin, Jung-Bae Kwon, Jong-Soo Kim, Jong-Gun Won, Jong-Sang Kim

**Affiliations:** 1 Division of Crops Research, Gyeongsangbuk-do Provincial Agricultural Research & Extension Services, Daegu 41404, Korea; tastypeach86@korea.kr (C.-M.H.); szzong91@korea.kr (J.-H.S.); borikae@korea.kr (J.-B.K.); jskim0429@korea.kr (J.-S.K.); ricewon@korea.kr (J.-G.W.); 2 Major in Food Biomaterials, School of Food Science & Biotechnology, Kyungpook National University, Daegu 41566, Korea

**Keywords:** crystallinity, gelatinization, pre-harvest sprouting, rice, rice flour, starch structure

## Abstract

Pre-harvest sprouting (PHS) severely reduces rice grain yield, significantly affects grain quality, and leads to substantial economic loss. In this study, we aimed to characterize the physicochemical properties and processing quality of the Garumi 2 flour rice variety under PHS conditions and compare them with those of the Seolgaeng, Hangaru, Shingil, and Ilpum rice varieties and the Keumkang wheat variety. Analysis of the molecular structure of starch revealed uniform starch granules, increased proportions of short-chain amylopectin in DP 6–12 (51.0–55.3%), and enhanced crystallinity (30.7–35.7%) in rice varieties for flour compared with the Ilpum cooking rice variety. PHS significantly altered the starch structure and gelatinization properties of Garumi 2. It also caused surface pitting and roughness in Garumi 2 starch granules and decreased their crystallinity. Collectively, the findings of this study provide important novel insights into the effects of PHS on the physicochemical properties of Garumi 2 floury rice for flour.

## 1. Introduction

Rice (*Oryza sativa* L.) is one of the three most important crops worldwide. It is a dietary staple for over 34% of the global population. Per capita rice consumption has declined by 2.3% annually over the past 30 years [1]. In contrast, wheat flour consumption has risen because of the increased consumer preference for fast food and other changes in dietary preference. Hence, food science research has investigated the replacement of wheat flour with rice flour in processed foods such as bread, cookies, cakes, and noodles [2,3].

To use rice flour as a raw material in processed foods, the source rice material must first be milled by the wet or dry method. The former entails soaking the rice in water and minimizes starch damage. It generates rice flour granules of uniform size and enhances product quality [4]. However, this technique involves extensive processing and incurs high wastewater treatment and drying costs [3]. Although dry milling is comparatively easy and cost-effective, it substantially damages rice flour starch. Therefore, the Korean government has developed novel rice varieties to produce a wheat flour substitute for the processed food industry. To date, several rice varieties for flour have been registered in Korea, such as Seolgaeng, Hangaru, Shingil, Mimyeon, Misiru, and Garumi 2. The latter has “floury endosperm (*flo7*)” that is easily ground in small-scale mills [5]. Seolgaeng yields milky, flavorful, non-glutinous rice grains conducive to brewing and dry milling for rice flour [6,7,8]. Hangaru was developed by hybridizing Daeripbyeo and Seolgaeng. The former bears large grains, whereas the latter is a high-quality Japonica generated by mutating fertilized ovules with *N*-methyl-*N*-nitrosourea (NMU) [6,7,8]. Shingil was derived from the mutation of Hanareum with NMU [9,10,11]. It has a soft endosperm and can be easily ground by dry milling.

Non-glutinous rice used in cooking is transparent and hard because its starch is compact. In contrast, rice varieties for flour are categorized as soft or floury. Floury rice starch granules are sparsely arranged throughout the endosperm, whereas soft rice starch granules are fine and arranged along the external region. In fact, soft and non-glutinous rice starch granules have similar appearance in endosperm cross-sections [12,13].

In the present study, Garumi 2 is a type of floury rice, while Seolgaeng, Hangaru, and Shingil are types of soft rice. Although these rice varieties for flour have excellent processing quality, little is known about their milling and other properties. The starch in rice varieties for flour has a molecular structure resembling that of wheat. However, polygonal rice varieties have unique starch structures amenable to dry milling and conducive to active pre-harvest sprouting (PHS).

PHS refers to seed germination before harvest. PHS occurs mainly under sustained high temperature and high relative humidity. PHS causes yield losses, adversely impacts grain quality, and results in considerable economic loss. Shingil has the lowest PHS ratios; those for Seolgaeng, Hangaru, Shingil, and Garumi 2 were 2.4%, 5.5%, 0.5%, and 23.2%, respectively. PHS significantly varied with transplanting date, nitrogen application, and climate [14]. Korea is endeavoring to maximize national rice flour yields. Hence, PHS-induced changes in rice flour starch warrant further research. In food processing, rice is generally used only in the preparation of rice cakes, alcoholic liquors, porridge, and confectionery. Rice cannot be easily used to prepare any other products and lacks differentiated materials. Thus, it is crucial to characterize the processing quality of rice flour to optimize it for use in processed food preparation. PHS-induced changes in rice starch physicochemistry and rice flour vulnerability must be investigated to facilitate the use of domestic rice flour as a raw material in processed food production.

The aims of this study were to elucidate the physicochemical properties of flour prepared by dry milling for five different rice varieties and to contribute to the development of novel rice utilization strategies in the processed food industry.

## 2. Materials and Methods

### 2.1. Rice Material and Experimental Design

The rice varieties for flour included Seolgaeng, Hangaru, Shingil, and Garumi 2. They were selected for comparison with the cooking rice variety Ilpum and the wheat variety Keumkang cultivated in Korea. In 2019, all five varieties were planted after tillage of a designated experimental plot at the Gyeongsangbuk-do Agricultural Research & Extension Services. The planting distance was 30 cm × 15 cm, and N-P_2_O_5_-K_2_O fertilizers were applied at the rates of 9–4.5–5.7 kg/10 a. Split fertilizer application was performed at basal (50%), tillering (25%), and panicle initiation (25%). When the grain moisture reached 15%, rice husks and impurities were removed with a laboratory rice huller (SY94+RAT2+2400; Ssangyong Machinery Industry Co., Ltd., Incheon, Korea), Garumi 2 was divided into Garumi 2-P (under PHS) and Garumi 2-N (non-PHS) by segregating samples with rice germs blackened by PHS. They were milled along with the other varieties, passed through a 100-mesh sieve, and used as test samples.

### 2.2. Starch Isolation

Rice grains were soaked in water, and their starch was isolated by alkaline treatment [15]. Soaked rice was dried, ground in a blender, and steeped in 0.2% (*w*/*v*) NaOH. The alkaline treatment was repeated until the yellow color disappeared and there was no further biuret reaction. The precipitates were collected, washed with deionized water, neutralized with 1 N HCl, washed again with deionized water, and centrifuged (VS-21SMT; Vision Scientific Co. Ltd., Daejeon-Si, Korea) at 1300× *g* and room temperature for 10 min. Isolated starch was dried at room temperature and passed through a 100-mesh sieve.

### 2.3. Color Measurement

To measure the color difference of a rice variety in its flour state, we put sample rice flours in a 30 mm transparent container and then used a colorimeter (JS-555; Color Techno System Co. Ltd., Yokohama, Japan) to measure Hunter’s L^*^ (lightness), ±a^*^ (redness/greenness), and ±b^*^ (yellowness/blueness) values three times to estimate the color difference. The cylindrical container was filled firmly to remove all air for sample measurement. The measurement was made at approximately <1 s, and the wavelength was 400–700 nm. A standard white plate was used after calibration. The color difference ΔE was calculated as $\sqrt{\left[{\left(\Delta L\right)}^{2}+{\left(\Delta a\right)}^{2}+{\left(\Delta b\right)}^{2}\right]}$ and was based on the white plate.

### 2.4. Granule Size Analysis

Granule size distribution of the flour from each experimental variety was measured using a laser diffraction particle size analyzer (Malvern Mastersizer 2000; Malvern Instruments Ltd., Malvern, UK). The samples of flour were immersed in ethanol for 30 s after ultrasonic treatment as previously described [16].

### 2.5. Damaged Starch Quantification

Damaged starch is the portion of starch that is mechanically disrupted during the processes used to extract or refine starch [17]. Damaged starch content was measured with a Megazyme kit (K-SDAM 02/2008; Megazyme International Ireland, Wicklow, Ireland) according to AACC Method No. 76-31. Rice flour (0.1 g) and fungal α-amylase were activated at 40 °C for 5 min. Thereafter, 1 mL (50 U/mL) fungal α-amylase was added to the rice flour and allowed to react at 40 °C for 10 min. Thereafter, 8 mL of 0.2% (*w*/*v*) sulfuric acid was added to terminate the reaction, and the mixture was centrifuged at 1000× *g* and room temperature for 5 min. Thereafter, 0.1 mL of each supernatant was transferred to a separate conical tube containing 0.1 mL amylose–glucosidase enzyme mixture (20 U/mL). The reaction proceeded at 40 °C for 10 min. Then, 4 mL GOPOD solution was added before a 20 min chromogenic reaction and absorbance was measured at 510 nm. The proportion of damaged starch was calculated as follows:

% damaged starch (*w*/*w*) = absorbance (sample) × (150/glucose standard absorbance)/sample weight (mg) × 8.1

### 2.6. Total Amylose and Protein Content

Rice endosperm amylose and protein were measured in a Cervitec Grain Inspector 1625 (Foss Analytical, Höganäs, Sweden). The Cervitec 1625 Grain Inspector used in our experiment is a device system that measures the amylose and protein contents of rice in a non-destructive manner. Milled rice samples were determined as previously described [18].

### 2.7. Starch Pasting Properties

Starch pasting properties were measured with a Rapid Visco Analyzer (RVA Model 4; Newport Scientific, Sydney, Australia). For each sample, 3 g rice flour and 25 mL deionized water were added to the sample canister and spun at 960 rpm for 10 s to produce rice flour suspensions. The mixtures were centrifuged at 160 rpm thereafter until the analysis was complete. The samples were heated to 50 °C for 1 min, and the temperature was increased at 12 °C/min until 95 °C, maintained at 95 °C for 2.5 min, cooled to 50 °C, and maintained at 50 °C for 2 min. The RVA viscogram plotted initial pasting temperature, and peak, trough, final, breakdown, and setback viscosities.

### 2.8. Differential Scanning Calorimetry

The gelatinization properties of starch were analyzed by differential scanning calorimetry (DSC) as previously described [19]. Briefly, 3.0 mg rice flour and deionized water were combined in a 1:2 (*v/v*) ratio, and the suspension was injected into an aluminum pan with a micro-syringe. The pan was sealed for 1 h and heated from 30 °C to 100 °C at 10 °C/min in a DSC (DSC 8500; PerkinElmer, Waltham, MA, USA). Onset temperature (T*_o_*), peak temperature (T*_p_*), conclusion temperature (T*_c_*), and gelatinization enthalpy (Δ*H*) were measured in triplicate.

### 2.9. Scanning Electron Microscopy

Starch granule and transverse section morphologies were compared by scanning electron microscopy (SEM). Grains were broken naturally and observed in cross-section by SEM (Mini-SEM SNE-4500M, SEC Co., Ltd., Suwon-si, Korea) as previously described [20]. Starch grains were coated with gold for conductivity and examined at 5 kV accelerating voltage, 100 s phototime, and 1000× and 5000× magnification with the same SEM.

### 2.10. Amylopectin Branch Chain Length Distribution

The amylopectin branch chain length distribution was analyzed in a high-performance anion-exchange chromatography system fitted with pulsed amperometric detection (HPAEC-PAD) according to a previously described method [21], with certain modifications [22]. The HPAEC system (Dionex DX500sem; Dionex Corp., Sunnyvale, CA, USA) consisted of a GP50 gradient pump, LC20-1 chromatography organizer, an ED40 electrochemical detector, a CarboPac PA-1 guard column (4 mm × 50 mm), a CarboPac PA-1 analytical column (4 mm × 250 mm), and an AS40 automated sampler [23]. Samples were passed through a filter with 0.45 μm pore size and separated by gradient elution from 100% eluent A (150 mM NaOH) to 100% eluent B (500 mM NaOAc in 150 mM NaOH). Samples were prepared from milled rice grains as previously described [23].

### 2.11. X-ray Diffraction and Crystallinity Analysis

The crystalline structure of the starches was analyzed by x-ray diffractometry (X’pert Pro MPD, Multi Purpose X-ray Diffractometer, PANalytical, Almelo, The Netherlands). Crystallinity and crystal strength were compared against the peak position and height measured at diffraction angles (2θ) in the range of 5–40° with a Cu-kα target. The scanning speed was 0.04° 2θ/s, the voltage was 40 kV, and the current was 20 mA. Relative crystallinity was divided into amorphous (Aa) and crystalline (Ac) regions and calculated with the formula (crystallinity (%) = Ac/(Aa + Ac) × 100) in Origin v. 7.0 (OriginLab, Northampton, MA, USA).

### 2.12. Fourier Transform Infrared Analysis

Fourier transform infrared (FT-IR) was used to examine the external regions of the starch granules [24]. The FT-IR spectrometer (Thermo Fisher Scientific Inc., Waltham, MA, USA) fitted with a MIR TGS detector was used to measure the FT-IR spectra for the rice starch granules. Each spectrum was measured at 1 cm^−1^ intervals in the range of 3500–1000 cm^−1^.

### 2.13. Statistical Analysis

One-way ANOVA was performed to identify significant differences among treatment means (*p* < 0.05). Duncan’s multiple range test was conducted to separate treatment means. The software used was Statistical Analysis System (SAS) v. 9.2 (SAS Institute, Cary, NC, USA). Data are presented as means ± standard deviation (SD) and all tests were conducted in triplicate.

## 3. Results and Discussion

### 3.1. Color Measurement

Rice flours color was reported in L* (lightness) for Hunter value, ±a* for red/green, and ±b* for yellow/blue (Table 1). Rice flour color generally differs with rice variety and starch granule size distribution. Overall, the rice varieties for flour examined herein displayed higher L (95.4–97.4) than Ilpum or Keumkang. Rice flour whiteness is an important flour quality determinant as consumers prefer white rice flour [25]. Garumi 2 especially exhibited high brightness value. These results were consistent with those of a previous report showing that floury rice had higher L* than soft rice [26]. The a* was often 0 or even <0 and near the green spectrum. Moreover, there were no differences among varieties. Ilpum rice had the lowest negative a*. This finding corroborated those of previous studies showing that hard rice granules typically have negative a* [26].

### 3.2. Granule Size Distribution

**Figure 1:**
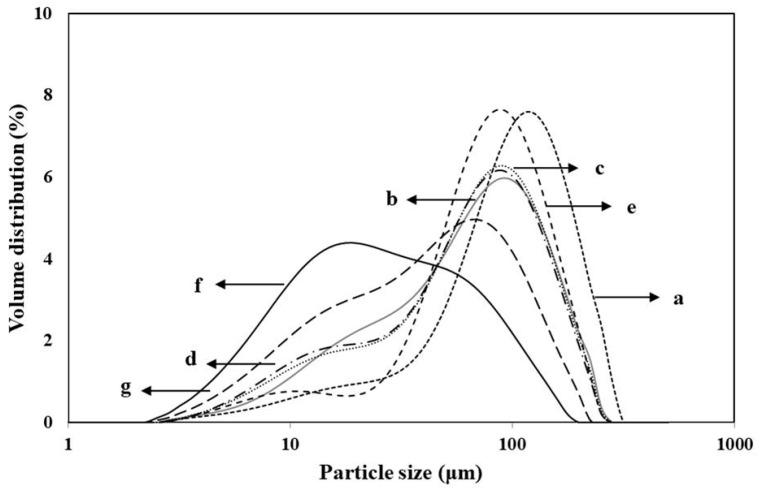
Particle size distribution of flours from six rice and one wheat varieties. (**a**) Ilpum, (**b**) Keumkang, (**c**) Seolgaeng, (**d**) Hangaru, (**e**) Shingil, (**f**) Garumi 2-P, and (**g**) Garumi 2-N. D_50_ = 50% of cumulative particle size distribution.

Ground rice flour granule size distribution directly affects final product (bread and noodle) quality as it influences gelatinization properties [27] and gel consistency [28,29,30,31,32]. Here, we compared rice flour granule size distribution by grinding all varieties and passing them through a 100-mesh sieve. Particle size distributions are shown in Figure 1. Ilpum exhibited the largest granule size (93.6 μm) and distribution (D_50_) >50%. In contrast, the other rice flours had granule sizes in the range of 40–73 μm and were ranked in the following order: Shingil > Seolgaeng > Keumkang > Hangaru > Garumi 2. The size of rice flour granule suitable for the production of Paeksolgi, a Korean traditional rice cake, is 60 mesh or 100 mesh [33]. Hence, the 60 mesh and 100 mesh granule sizes of the rice varieties examined herein are appropriate for quality food product processing. However, the granules of Garumi 2-P were only 22.9 μm and were much smaller than those of Garumi 2-N. Therefore, Garumi 2-P is unsuitable for preparing rice cakes that are highly popular in Korea. The fine granules of Hangaru and Garumi 2 might explain the increased L* and decreased b* of these varieties.

### 3.3. Damaged Starch Quantification

Table 1 summarizes the percentage of damaged starch for different rice varieties compared with Keumkang wheat. There was ~8% starch damage in Ilpum and ~4%–6% starch damage in the other rice varieties. Garumi 2-N had the lowest starch damage content. Damaged starch may serve as a starch hydrolase substrate, but excess starch negatively affects dough quality. The interface between air and dough might be unstable during dough production. It may reduce rising and result in poor bread crumb quality because of internal gas cell adhesion during baking [34]. Rice flours with fine granule size had high level of damaged starch [35]. The starch damage of rice flour is correlated with granule size. Rice flours with fine granules are not suitable for high-quality food product processing as they have a high damaged starch content [4]. Nevertheless, even the rice varieties with fine granules may have low damaged starch content if there is minimal starch damage during the milling process. There was more severe starch damage in Garumi 2-P than Garumi 2-N. Furthermore, the former had poor processing quality.

### 3.4. Total Amylose and Protein Content

The main rice endosperm components related to palatability are amylose and protein contents. Palatability increases when the protein content is <7% and the amylose content is low. Protein content is correlated with rice hardness [36,37]. The protein is concentrated in the rice endosperm surrounding starch particle cell membranes. The starch and protein form a complex. As the cell membrane is hard, it is resistant to thermal degradation. However, the rheological properties of the starch gel are impaired [38,39]. The milled rice with low amylose content undergoes minimal expansion during cooking. After cooking, this type of rice grain is glossy, sticky, and relatively firm [40].

The protein and amylose contents were measured with a Cervitec grain inspector. The protein content differed by ≤0.5% among Ilpum, Seolgaeng, Hangaru, and Shingil. Garumi 2 showed a significantly lower protein content than the other varieties but the protein content was higher in Garumi 2-P than Garumi 2-N. Shingil and Garumi 2 had the highest and lowest protein content, respectively. The amylose content was similar in Shingil and Keumkang. Both varieties displayed the highest amylose content among all six strains tested. All rice varieties for flour except Shingil contained lower amylose content than Ilpum. Garumi 2-P exhibited less amylose than Garumi 2-N (Table 1). This result was in agreement with several studies on sprouted rice [41,42,43].

### 3.5. Pasting Properties

Pasting is the phenomenon following gelatinization in the dissolution of starch. It involves granular swelling, exudation of molecular components from the granule, and eventually, total disruption of the granules [17]. Rice starch pasting properties were variable depending on rice variety (Table 2). Japonica varieties with high taste values have low pasting temperatures, high peak and breakdown viscosity, and low final viscosity. High amylose content decreases peak and breakdown viscosities and increases setback viscosity [44]. Shingil exhibited the lowest peak, trough, final, and breakdown viscosities and the highest setback viscosity compared with the other varieties. All varieties for flour except Shingil displayed higher breakdown viscosity than Ilpum. For Garumi 2-P and Garumi 2-N, the peak viscosity was higher than the final viscosity, while the highest breakdown and setback viscosities were negative. Garumi 2-P had higher peak and trough viscosities than Garumi 2-N. Shingil showed the highest pasting temperature, followed by Seolgaeng and Hangaru. In contrast, the pasting temperatures for Garumi 2 and Ilpum were in the range of 72–74 °C. In this study, Garumi 2-P exhibited high viscosity parameters in comparison to Garumi 2-N. As shown in Figure 1, the distribution of smaller particle sizes of Garumi 2-P was higher than that of Graumi 2-N. This effect was associated with a faster swelling of the starch granules in PHS flours due to floury endosperm. This result was in agreement with the findings of Zhang [45] who reported that floury endosperm flour showed higher swelling ability.

### 3.6. Thermal Properties

DSC thermodynamically describes gelatinization by measuring the enthalpy of the endothermic starch gelatinization reaction. DSC measures heat absorption or release by chemical reactions during phase changes such as melting [46]. DSC characteristics of the rice varieties for flour are shown in Table 3. Shingil had the lowest gelatinization temperature according to DSC and BD in the RVA measurement, suggesting that Shingil had low heat transfer resistance during gelatinization. Hangaru exhibited the highest gelatinization enthalpy, and thus enthalpy relatively more energy to melt starch crystal. Garumi 2-P displayed a higher gelatinization temperature and enthalpy than Garumi 2-N.

### 3.7. Morphological Studies by SEM

**Figure 2:**
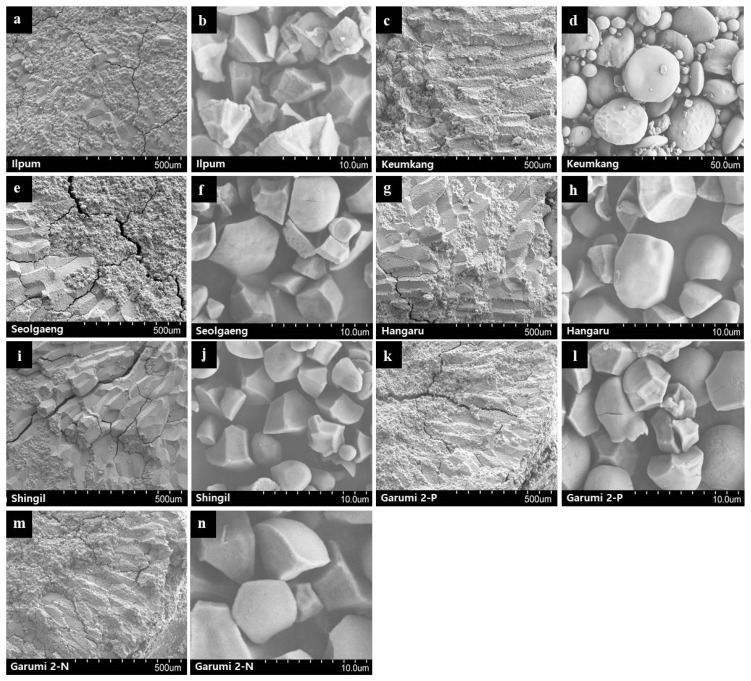
SEM of flour and starch granules isolated from six rice and one wheat varieties. (**a**,**b**) Ilpum, (**c**,**d**) Keumkang, (**e**,**f**) Seolgaeng, (**g**,**h**) Hangaru, (**i**,**j**) Shingil, (**k**,**l**) Garumi 2-P, and (**m**,**n**) Garumi 2-N.

The starch samples from 5 rice and Keumkang wheat varieties were morphologically compared by SEM (Figure 2). In general, rice starch granules are polygonal, whereas wheat starch granules are round; moreover, the former are harder than the latter. The flours derived from the rice varieties examined herein had round starch granules, which is similar to that of wheat because the rice varieties tested here were specifically developed to replace wheat flour. All rice varieties showed rounder starch granules than those of Ilpum but were not perfectly round like wheat starch granules. Garumi 2 and Hangaru had the roundest starch granules, followed by Seolgaeng. In contrast, Ilpum starch granules were irregular polygons. Starch granules of Garumi 2-N were smooth, whereas those of Garumi 2-P were pitted (Figure 2). Ilpum starch granules were harder and more uniform than those of the other varieties. For the soft rice varieties (Seolgaeng, Shingil, and Hangaru), the external endosperm region was smooth, and the interior was filled with debris. For the floury rice variety (Garumi 2), debris was dispersed throughout the endosperm. Nonetheless, the debris content was lower in floury variety than soft rice. Floury rice might readily degrade as its granule sizes are uniform and its damaged starch content is low. Thus, floury rice is appropriate for milling. Garumi 2-P was softer than Garumi 2-N, possibly because the former contained uneven debris.

### 3.8. Amylopectin Branch Chain Length Distribution

Table 4 shows comparison of the amylopectin chains in the rice varieties with those in Keumkang wheat. Based on the degree of polymerization (DP), the amylopectin branch chains were classified as A (DP 6–12), B1 (DP 13–24), B2 (DP 25–36), and B3+ (DP ≥ 37) [47]. Significant variations were observed among the varieties in terms of gelatinization temperature as the amylopectin microstructure affected the gelatinization properties. The heat requirement increased in proportion to the content of short amylopectin chain because this structure had a high gelatinization temperature [48]. In Keumkang wheat flour, the proportion of amylopectin with DP ≥ 10 was >85%, and there were few short chains. In Shingil rice, the proportion of amylopectin with DP 6–12 was ≥50%. This finding was consistent with that of a previous study [49] (Table 4). The short-chain amylopectin distribution was more uniform in Garumi 2-N than Garumi 2-P. Thus, they differed in starch crystallinity.

### 3.9. X-ray Diffraction and Crystallinity

**Figure 3:**
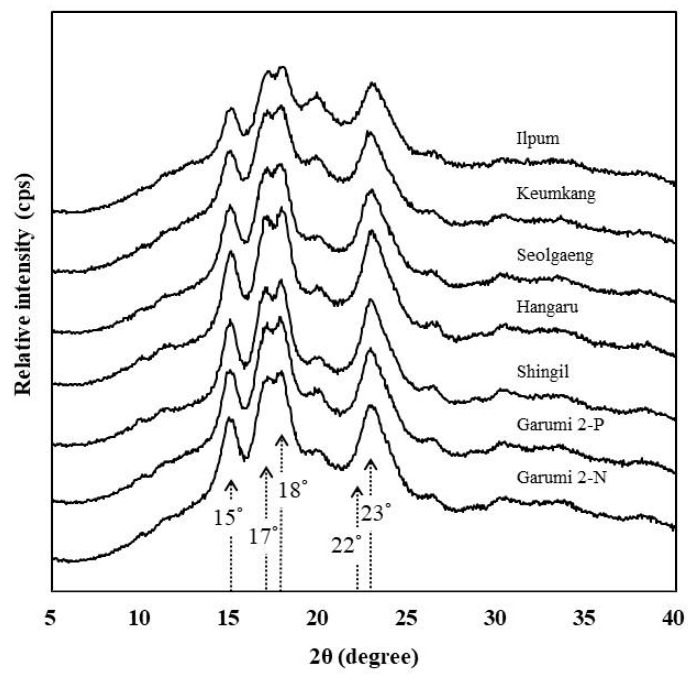
X-ray diffraction (XRD) patterns of starches in six rice varieties and one wheat variety for flour.

X-ray diffraction (XRD) patterns of the rice varieties were compared from each other in terms of starch granule crystallinity. This parameter was also used to analyze amylopectin crystallinity. The diffraction width was narrow and sharp as crystallinity increased. Hence, the starch granule clusters were regular [49]. Both wheat and rice starch granules showed strong peaks at the 15°, 17–18°, and 22–23° diffraction angles. These data correspond to an A-type peak pattern for starch, which is characterized by a dense structure and low moisture content [50]. Thus, there was no change in rice starch crystal structure (Figure 3). Relative crystallinity was estimated from the ratio of the peak areas to the total diffractogram area. The latter is the sum of the peak and amorphous areas [51]. The Ilpum had a relatively lower degree of crystallinity than wheat and the other rice varieties (Table 4). Moreover, the degree of crystallinity was slightly lower for Garumi 2-P than Garumi 2-N. Seolgaeng displayed the sharpest peak and highest degree of crystallinity.

### 3.10. FT-IR Spectroscopy

**Figure 4:**
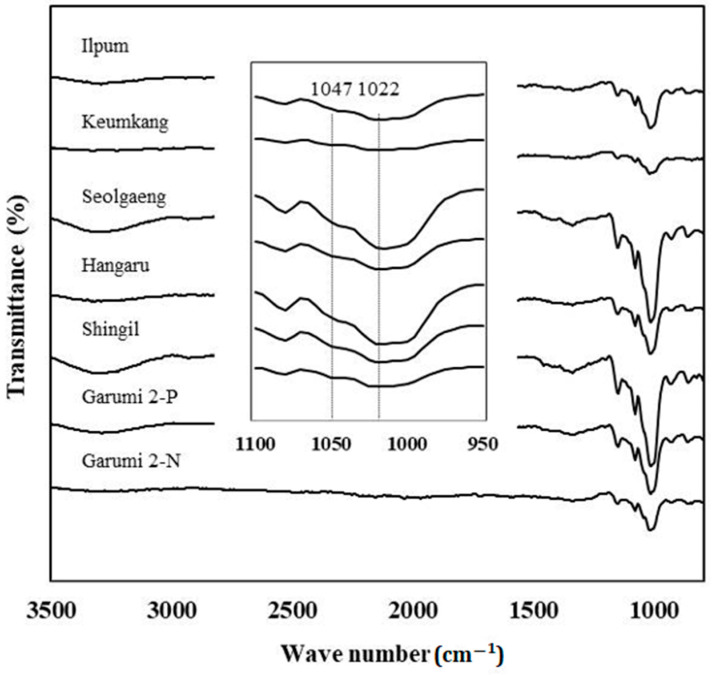
FT-IR spectra of starches from six rice varieties and one wheat variety for flour.

FT-IR spectroscopy is a useful and sensitive tool for qualitatively and quantitatively analyzing processed foods. It also identifies starch crystal structure within specific spectra and characterizes their functional groups. The comparison of the FT-IR spectra for the rice varieties against that for wheat disclosed three peaks, indicating pyranose in the 1014 cm^−1^/1155 cm^−1^ ratio. There was also OH stretching at 3200 cm^−1^/3500 cm^−1^, OH bending at 1580 cm^−1^/1640 cm^−1^, and CH bending at 1330 cm^−1^/1400 cm^−1^ [52,53]. There were only slight variations in the peak strengths of each area, and all rice varieties displayed similar patterns (Figure 4). The peak intensity ratio at 1047 cm^−1^/1022 cm^−1^ is employed to determine the degree of crystallinity of the starch granule surfaces [24]. In the present study, Garumi 2-P showed a lower ratio (1047 cm^−1^/1022 cm^−1^) than Garumi 2-N. This result was consistent with the XRD data (Table 4). The 1047 cm^−1^/1022 cm^−1^ ratio mainly reflects the starch double-helix content. Thus, the FT-IR results indicated that PHS had a slight alteration in the double-helical alignment of the starch granule region. These results are consistent with a previous study that germination reduced the 1045/1022 ratio [53].

## 4. Conclusions

The present study investigated the physicochemical properties of five rice varieties for flour. It demonstrated relative differences between the physicochemical properties of Garumi 2 under PHS and non-PHS conditions. The rice varieties for flour had lower starch damage rates (<6%), smaller granules (40–73 μm), and more uniform starch granule surfaces than the cooking rice variety Ilpum. The RVA analysis revealed that Shingil had the lowest viscosity. The thermal enthalpy of gelatinization was lowest for Shingil and highest for Hangaru, whereas the short-chain amylopectin ratio was highest for Shingil. The XRD analysis demonstrated that the relative degree of crystallinity for Shingil closely approached that of Keumkang. In contrast, Ilpum had the lowest degree of crystallinity. The FT-IR analysis showed that the 1047/1022 cm^−1^ ratio was highest for Garumi 2 and lowest for Ilpum.

The results of this study suggested that Garumi 2 had superior processing quality as characterized by uniform starch granules, high heat transfer resistance, low starch damage rates, and starch granules similar in size to that of wheat flour. Moreover, this research revealed that PHS caused severe starch damage, an excess of small granules, an increase in pasting properties, alterations in short-chain amylopectin composition, changes in starch structure, and a reduction in the degree of crystallinity in Garumi 2. Overall, the findings of this study provide useful insights into the effects of PHS on rice starch physicochemistry and may contribute to the development of novel rice strains suitable for the food processing.

## Figures and Tables

**Table 1:** Physicochemical characteristics of the flours obtained from different varieties of rice.

Variety	Amylose Content (%)	Protein Content (%)	Damaged Starch (%)	Color Value			
				L*	a*	b*	ΔE
Ilpum	19.5 ± 1.06 ^b^	7.8 ± 0.12 ^c^	8.1 ± 0.37 ^b^	94.61 ± 0.04 ^e^	−0.76 ± 0.01 ^g^	6.50 ± 0.01 ^b^	8.00 ± 0.02 ^b^
Keumkang **	22.3 ± 1.55 ^a^	12.5 ± 0.45 ^a^	9.5 ± 0.37 ^a^	92.75 ± 0.16 ^f^	0.61 ± 0.03 ^a^	8.48 ± 0.17 ^a^	10.48 ± 0.21 ^a^
Seolgaeng	17.3 ± 0.08 ^b^	7.7 ± 0.53 ^cd^	5.2 ± 0.16 ^d^	95.94 ± 0.02 ^c^	−0.17 ± 0.01 ^f^	4.48 ± 0.02 ^c^	5.70 ± 0.01 ^c^
Hangaru	17.6 ± 0.69 ^b^	8.0 ± 0.16 ^bc^	5.7 ± 0.16 ^c^	95.94 ± 0.14 ^c^	0.06 ± 0.01 ^d^	3.79 ± 0.02 ^e^	5.02 ± 0.01 ^d^
Shingil	22.2 ± 1.55 ^a^	8.2 ± 0.24 ^b^	5.1 ± 0.24 ^d^	95.40 ± 0.18 ^d^	0.00 ± 0.02 ^e^	4.14 ± 0.11 ^d^	5.48 ± 0.16 ^c^
Garumi 2-P	18.8 ± 0.16 ^b^	7.3 ± 0.37 ^de^	5.0 ± 0.08 ^d^	97.39 ± 0.17 ^a^	0.28 ± 0.01 ^b^	1.66 ± 0.06 ^g^	2.87 ± 0.04 ^f^
Garumi 2-N	19.4 ± 0.91 ^b^	7.0 ± 0.16 ^e^	4.4 ± 0.00 ^e^	96.65 ± 0.25 ^b^	0.18 ± 0.01 ^c^	2.82 ± 0.05 ^f^	3.98 ± 0.06 ^e^

Data are presented as mean of three determinations. Different letters within the same column indicate significant differences according to Duncan’s multiple range test (*p* < 0.05). L*, lightness/darkness value; a*, red/green axis; b*, yellow/blue axis; ΔE, total color difference value. ** wheat variety.

**Table 2:** Pasting properties of flours obtained from different varieties of rice.

Variety	Pasting Time (min)	Pasting Temp (°C)	Viscosity (cP)				
			PV	HPV	CPV	BD	SB
Ilpum	2.9 ± 0.03 ^e^	72.6 ± 0.40 ^e^	2765 ± 35.12 ^b^	2190 ± 17.21 ^a^	3274 ± 28.58 ^a^	575 ± 25.35 ^d^	508 ± 17.52 ^c^
Keumkang *	4.4 ± 0.07 ^b^	90.3 ± 0.73 ^b^	1167 ± 33.73 ^d^	898 ± 22.84 ^e^	1768 ± 45.70 ^e^	269 ± 11.78 ^e^	601 ± 20.61 ^b^
Seolgaeng	4.0 ± 0.03 ^c^	84.9 ± 0.38 ^c^	2374 ± 12.66 ^c^	1534 ± 41.04 ^c^	2652 ± 44.84 ^c^	840 ± 30.27 ^b^	277 ± 41.71 ^e^
Hangaru	3.9 ± 0.00 ^c^	84.6 ± 0.05 ^c^	2430 ± 41.35 ^c^	1664 ± 74.37 ^b^	2832 ± 72.69 ^b^	766 ± 35.95 ^c^	402 ± 31.38 ^d^
Shingil	4.8 ± 0.06 ^a^	94.3 ± 0.82 ^a^	646 ± 19.34 ^e^	548 ± 15.77 ^f^	1318 ± 36.74 ^f^	98 ± 11.90 ^f^	671 ± 25.49 ^a^
Garumi 2-P	3.1 ± 0.03 ^d^	74.6 ± 0.40 ^d^	3091 ± 46.48 ^a^	1692 ± 30.92 ^b^	2896 ± 45.96 ^b^	1398 ± 19.19 ^a^	−195 ± 2.49 ^f^
Garumi 2-N	3.0 ± 0.06 ^de^	73.6 ± 0.67 ^de^	2723 ± 74.68 ^b^	1387 ± 17.05 ^d^	2528 ± 36.46 ^d^	1336 ± 58.79 ^a^	−195 ± 39.42 ^f^

Data are presented as means of three determinations. Different letters within the same column indicate significant difference according to Duncan’s multiple range test (*p* < 0.05). PV, HPV, and CPV are peak, hot paste, and cool paste viscosities, respectively. BD and SB are breakdown and setback viscosities, respectively. Breakdown viscosity is the difference between peak viscosity and the holding strength or hot paste viscosity. Consistency is the difference between the final (cool paste) viscosity and the holding strength. Setback is the difference between the final and peak viscosities. * wheat variety.

**Table 3:** DSC characteristics of flours obtained from different varieties of rice.

Variety	T*_o_* (°C)	T*_p_* (°C)	T*_c_* (°C)	ΔT (T*_c_*-T*_o_*)	Δ*H* (J/g)
Ilpum	63.98 ± 0.33 ^bc^	69.49 ± 0.10 ^b^	74.56 ± 0.20 ^b^	10.58 ± 0.34 ^b^	8.11 ± 0.71 ^ns^
Keumkang *	62.10 ± 1.02 ^d^	65.16 ± 0.38 ^d^	69.46 ± 0.79 ^e^	7.35 ± 1.79 ^c^	6.36 ± 0.72
Seolgaeng	63.07 ± 0.29 ^cd^	68.07 ± 0.30 ^c^	72.74 ± 0.22 ^c^	9.67 ± 0.34 ^b^	8.06 ± 2.30
Hangaru	63.79 ± 0.07 ^bc^	68.53 ± 0.21 ^c^	73.40 ± 0.37 ^c^	9.61 ± 0.39 ^b^	9.41 ± 1.50
Shingil	58.56 ± 0.43 ^e^	64.08 ± 0.42 ^e^	71.45 ± 0.79 ^d^	12.89 ± 0.36 ^a^	7.20 ± 2.45
Garumi 2-P	64.80 ± 0.15 ^a^	70.90 ± 0.19 ^a^	75.95 ± 0.12 ^a^	11.15 ± 0.19 ^b^	8.65 ± 0.75
Garumi 2-N	65.09 ± 0.14 ^ab^	70.68 ± 0.07 ^a^	75.25 ± 0.21 ^ab^	10.16 ± 0.12 ^b^	7.50 ± 0.39

Data are presented as means of three determinations. Different letters within the same column indicate significant difference according to Duncan’s multiple range test (*p* < 0.05). T*_o_*, T*_p_*, and T*_c_* are the onset, peak, and conclusion temperatures of gelatinization, respectively. ΔT(T*_c_*–T*_o_*) is the gelatinization temperature range. Δ*H* is the gelatinization enthalpy change. * wheat variety. ns represents ‘no statistical significance’.

**Table 4:** Comparison of the chain length distribution of amylopectin, relative crystallinity, and IR parameters of flours obtained from different varieties of rice under study.

Variety	Distribution (%)				Relative Crystallinity (%)	1047 cm^−1^/1022 cm^−1^ IR Ratio
	DP 6–12	DP 13–24	DP 25–36	DP > 37		
Ilpum	50.78 ± 0.02 ^b^	31.32 ± 0.02 ^b^	3.80 ± 0.02 ^b^	0.49 ± 0.01 ^b^	25.0 ± 0.41 ^e^	1.03 ± 0.00 ^f^
Keumkang *	42.80 ± 0.76 ^d^	47.38 ± 0.82 ^a^	8.17 ± 0.15 ^a^	0.69 ± 0.01 ^a^	35.0 ± 0.37 ^a^	1.02 ± 0.00 ^f^
Seolgaeng	50.99 ± 0.06 ^b^	31.53 ± 0.08 ^b^	3.63 ± 0.05 ^c^	0.45 ± 0.01 ^c^	35.7 ± 0.57 ^a^	1.12 ± 0.00 ^d^
Hangaru	50.90 ± 0.15 ^b^	31.62 ± 0.09 ^b^	3.61 ± 0.08 ^c^	0.45 ± 0.00 ^bc^	30.7 ± 0.33 ^d^	1.07 ± 0.00 ^e^
Shingil	55.32 ± 0.16 ^a^	30.46 ± 0.12 ^c^	3.15 ± 0.04 ^d^	0.25 ± 0.03 ^e^	33.5 ± 0.16 ^b^	1.27 ± 0.01 ^a^
Garumi 2-P	48.02 ± 0.96 ^c^	27.23 ± 0.50 ^e^	3.27 ± 0.02 ^d^	0.47 ± 0.02 ^bc^	31.9 ± 0.12 ^c^	1.22 ± 0.01 ^c^
Garumi 2-N	51.88 ± 0.58 ^b^	28.08 ± 0.23 ^d^	2.68 ± 0.05 ^e^	0.38 ± 0.00 ^d^	32.8 ± 0.16 ^b^	1.25 ± 0.01 ^b^

Data are presented as means of three determinations. Different letters within the same column indicate significant difference according to Duncan’s multiple range test (*p* < 0.05). Sum of peak-area ratios (%) with degree of polymerization (DP). Relative crystallinity was calculated by dividing it into amorphous (Aa) and crystalline (Ac) regions. (Crystallinity (%) = Ac/(Aa + Ac) × 100). * wheat variety.

## Data Availability

Not applicable.
